# ‘Birds of the same feathers fly together’: midwives’ experiences with pregnant women and FGM/C complications - a grounded theory study in Liberia

**DOI:** 10.1186/s12978-019-0681-1

**Published:** 2019-02-14

**Authors:** Christine K. Tarr-Attia, Grace Hawa Boiwu, Guillermo Martínez-Pérez

**Affiliations:** 1 Saint Joseph’s Catholic Hospital, Monrovia, Liberia; 2 African Women’s Research Observatory (AfWORO), Barcelona, Spain; 3 NGO Nutrition Without Borders The Gambia, Centre for Rehabilitation and Education in Nutrition, Basse Santa Su, URR The Gambia; 4 Faculty of Health Sciences, University of Saragossa, Saragossa, Spain

**Keywords:** Female genital mutilation/cutting, Female circumcision, Grounded theory, Qualitative research, Liberia, Midwives

## Abstract

**Background:**

In Liberia, approximately 70% of the women of the North-Central and North-Western regions could have undergone female genital mutilation/cutting (FGM/C) in their childhood during a traditional ceremony marking their entrance into Sande, a secret female society. Little is known about FGM/C from Liberian women’s perspective. This study aimed to understand the health implications of FGM/C as perceived by qualified female midwives.

**Methods:**

This qualitative study was conducted in 2017 in Monrovia, Liberia’s capital. Twenty midwives were approached. Of these, seventeen consented to participate in in-depth interviews. A thematic guide was used to gain insights about their knowledge on FGM/C and their experiences attending women victims of FGM/C. A feminist interpretation of constructivist grounded theory guided data generation and analysis.

**Results:**

The midwives participants described how clitoridectomy was the most common FGM/C type done to the girls during the Sande initiation ceremonies. Sexual impairment and intrapartum vulvo-perineal laceration with subsequent hemorrhage were described as frequent FGM/C-attributable complications that some midwives could be unable to address due to lack of knowledge and skills. The majority of midwives would advocate for the abandonment of FGM/C, and for the preservation of the traditional instructions that the girls in FGM/C-practicing regions receive when joining Sande. The midwives described how migration to urban areas, and improved access to information and communication technologies might be fuelling abandonment of FGM/C.

**Conclusion:**

Liberian midwives need tailored training to provide psychosexual counseling, and to attend the obstetric needs of pregnant women that have undergone FGM/C. In spite of FGM/C being seemingly in the decline, surveillance at clinic-level is warranted to prevent its medicalization. Any clinic- or community-based training, research, prevention and awareness intervention targeting FGM/C-practicing populations should be designed in collaboration with Sande members, and acknowledging that the Liberian population may place a high value in Sande’s traditional values.

## Plain English summary

Female genital mutilation/cutting (FGM/C) is a practice that harms the health of women and girls who undergo it. In Liberia, FGM/C is done to young girls when they attend the Sande initiation ceremonies. The Sande is a secret female society of ancestral origin. In 2017, to better understand Liberian health professionals’ perspectives on the health consequences of FGM/C, seventeen female midwives were interviewed in Monrovia, Liberia’s capital. Using a feminist approach to qualitative data generation, the interviews held were helpful to understand what midwives’ experiences attending women victims of FGM/C in their consultations were. The midwives described how cutting of the clitoris was especially common in the North-Central and North-Western counties of Liberia. Its main purpose was to inhibit women’s sexual pleasure and desire. Sexual dysfunction and vulvo-perineal laceration and hemorrhage during delivery were described as its most common complications. The majority of midwives would advocate for the abandonment of FGM/C. However, all but one midwife would appreciate if the girls could continue receiving the moral teachings that girls receive during the initiation ceremonies. Even in the absence of an anti-FGM/C legislation, migration to Monrovia and access to communication technologies might be helping mothers decide not to subject their daughters to FGM/C. In conclusion, Liberian midwives need to receive training to improve healthcare provision to women victims of FGM/C. Because a traditional sector of the Liberian society may appreciate Sande and its moral values, any intervention on FGM/C health complications should be designed in collaboration with FGM/C-practicing communities.

## Introduction

More than 200 million women globally have suffered Female Genital Mutilation/Cutting (FGM/C) [1]. The World Health Organization (WHO) classes FGM/C in three excisional types: clitoridectomy (Type I), excision (Type II) and infibulation (Type III) [1]. All these types may cause harmful gynaeco-obstetric consequences. Dyspareunia, bacterial vaginosis, impaired sexual function, and related psychological disorders are long-term consequences associated to any FGM/C type [2–5]. During pregnancy, women who suffered FGM/C are at increased risk of urinary tract infections and preterm labor [6]. Women also have higher risk of prolonged labor, severe vaginal-perineal trauma in vaginal delivery [7], and of delivery by caesarean section [8, 9].

There is concern about the persistence of FGM/C [10, 11]. In many countries, such as Kenya or Nigeria, in spite of anti-FGM/C legislation, medicalization of FGM/C is common [12–14]. In Liberia, where there is a lack of legislation against FGM/C [14], approximately 70% of the women of the North Western and North Central regions have undergone FGM/C as a ritual prerequisite to Sande membership [15].

The Sande is an ancient women-only society that plays a significant economic, political, social, and educational role in Liberia. The existence of Sande, and of the practice of clitoridectomy (FGM/C Type I) in its initiation ceremony, is known since the XVII^th^ Century [16, 17]. The Sande, and the Poro –its male equivalent, are also present in Sierra Leone (i.e. known as the Bondo or Bundu society) and in Guinea Conakry [18–21]. Both Sande and Poro place emphasis on secrecy in all their ritual and organizational activities. Women are initiated into Sande before or around puberty. However, their participation in Sande continues throughout their lives.

Scholar Fuambai Ahmadu recalled her insider perspectives as a Sierra Leonean woman who experienced FGM/C [18]. As Ahmadu put it, the *‘unfortunate and perturbing silence among African women intellectuals who have experienced initiation’* may explain the dearth of Liberian women’s voices on FGM/C. The secrecy surrounding Sande’s rituals may explain why there is paucity of clinical and epidemiological information on FGM/C in Liberia. The 2007 and 2013 Demographic Health Surveys queried about women’s membership to Sande, but did not ask on their actual FGM/C status [15, 22].

To improve healthcare provision to victims of FGM/C, the knowledge, attitudes and practices of healthcare professionals must be explored [23]. The purpose of this study is to understand the health implications that may be associated to FGM/C, as perceived and narrated by Liberian registered midwives. Other secondary objectives are to understand the motivations that support the practice, to document how midwives manage FGM/C complications, and to define the training needs for Liberian midwives. This information will be helpful for health policy-makers and health education providers to inform the design of culture-sensitive training, clinical and community awareness programs for the regions in Liberia where the Sande and Poro operate [23].

## Materials and methods

### Design and site

This was a qualitative study that was conducted in Monrovia, Liberia’s capital, in 2017, and targeting registered midwives as study population.

In Liberia, pregnant women may receive free-of-charge pregnancy care in government-run clinics. At community-level, traditional birth attendants (TBA) can accompany women during their pregnancy, but must refer them for delivery to their nearest clinic. At clinic-level, mothers may be attended by registered midwives or –in the absence of midwives–, by nurses. Since 2013, midwives can register as trained obstetric clinicians at the Liberian Medical and Dental Council [24]. The last DHS reported that approximately 61% of births had been attended by skilled birth attendants [15]. In spite of progresses in access to maternal health services, the utilization of clinic-based pregnancy care services significantly reduced during the 2014–16 Ebola outbreak in Liberia [25, 26].

### Recruitment

Female registered midwives born in Liberia; knowledgeable of FGM/C; and who had worked as midwives for at least two years were targeted as study participants. One member of the research team, a female midwife with experience in qualitative research, was the recruiting agent and interviewer.

Purposive sampling was used to approach the first two midwives participants. The recruiting agent purposively approached two midwives who were randomly selected from a list of trainees in a workshop on domestic violence that had been previously led by her. Purposive sampling continued from these first two participants, who recommended other potential participants to the recruiting agent.

All midwives were contacted telephonically and invited to participate in an in-depth interview (IDI) in their location of choice within Monrovia. At the scheduled date, written informed consent was obtained prior to the start of the IDI.

Recruitment was to continue until data saturation was gained. Nevertheless, recruitment was prematurely stopped due to the refusal to participate of three midwives who disclosed being Sande, and who manifested disagreement with the study. At that point, for security reasons, the research team decided to terminate sampling of participants.

### Data management and analysis

All IDIs were conducted in ‘colloquia’ (i.e. Liberian English) using a broad thematic or topics guide that aimed to gain insights about the midwives’ perceptions and knowledge on FGM/C, and about their experiences attending pregnant women who had undergone FGM/C. IDI recordings were transcribed verbatim into a MS Word. All transcriptions were cross-checked against the recordings. The final transcripts were imported into Dedoose software (®SocioCultural Research Consultants, Manhattan Beach, CA).

A feminist interpretation of constructivist grounded theory was considered the most appropriate methodological approach to engage midwives in a study on practices that are harmful to women’s health [27–29]. In practical terms, most methodological decisions were taken in agreement with best guidance on constructivist grounded theory research conduct [27–29]. Hence, data generation, coding and analysis happened contemporaneously. As data analysis began whilst data generation was ongoing, an in-depth exploration of the themes that the participants referred to as especially relevant was possible. Data was first line-by-line coded in a printed set of transcriptions. Once a coding framework was developed based on concepts and categories emerging during the first five IDIs, this framework was used to recode the first transcripts and to code the following IDIs using Dedoose.

To ensure trustworthibility, upon finalization of each IDI, the interviewer and the principal investigator jointly analyzed its transcription to detect recall and social desirability bias; discussion topics that seemed overly sensitive; and emerging themes that deserved more thorough discussion. Participants’ answers from the IDIs were triangulated with reports from the literature. At the end of the analysis phase, the study findings were presented at the University of Liberia institutional review board.

During the whole data generation, analysis and reporting processes, the feminist interpretation involved that: i) the midwives were addressed as ‘co-interpreters’ of the meanings of the study findings in cooperation with the interviewer; ii) the interviewer (a woman) reflected upon the impact of her own characteristics on the midwives during her interactions with them; iii) the research team (two women, one man) was sensitive towards issues of oppression that might be affecting the participants; iv) the research team ensured that the findings were useful to promote social change, and, hence, that vi) the research team promoted local dissemination to translate findings into policy, healthcare and training practice change.

### Ethics

Informed consent was sought for all participants. During the consent process, each participant was clearly informed about the study purpose; the organizations involved; the potential risk of social harm that could derive from their participation; their right to withdraw from the study at any time; the measures in place to protect their privacy and confidentiality. All participants received a signed copy of all consent documents.

Strict measures to minimize the possibility of social harm were taken. To protect the participants’ privacy, interviews were held at a location of their choice, and no researcher other than the recruiting midwife met any of the participants. To prevent discomfort, participants were reassured that they did not have to answer any question they do not wish to, and that no questions on their own history with FGM/C were to be asked.

To ensure confidentiality, socio-demographics were collected only if consent for such data to be registered was given. The consent forms were the only documents where the participants’ full names were included. No personal identifier from any research documents was captured in any database. Personal identifiers were removed from the IDI transcriptions. To further prevent unwanted identification of the participants, disaggregated socio-demographic data are not reported in this article, and all IDI recordings were deleted once the analysis on their transcriptions ended.

Ethics approval was obtained from the University of Liberia-Pacific Institute for Research and Evaluation Institution Review Board.

## Results

### Socio-demographic characteristics

Seventeen midwives consented to participate. Three midwives refused. The median age of the participants was 49 years (Range 38–63). In average, they had worked as midwives for 16 years (Range 5–37). Ten of them had completed both midwifery and nursing studies. Five had never worked in rural areas. All were Christian of different denominations. Ten were born in counties where, according to the 2013 DHS [15], the Sande society operates (i.e. Grand Bassa, Lofa, Montserrado and Nimba counties). During the interviews, only three disclosed that they were Sande members. Recordings lasted a mean of 50 (Standard Deviation 14.68) minutes.

Throughout this Results section, to better reflect the midwives’ viewpoints, we refer to key concepts using their own words in *‘italics’*. Interpretations of the phenomena under study derive from their narratives only. Unless stated otherwise, reported insights were common throughout all IDIs. The subheadings below correspond to the core themes that were inductively identified during the data analysis.

### Describing traditional interventions on the genitalia

All midwives agreed that clitoridectomy (i.e. FGM/C Type I) was commonly practiced in the country’s Northern and Western counties. About half of the midwives mentioned that they had attended some pregnant women whose labia minora were also cut (i.e. in reference to FGM/C Type II). Throughout the interviews, the terms female genital mutilation, female genital cutting (FGC), and female circumcision were used. FGC was the term that most midwives preferred. However, the use of any of these three terms could be inappropriate in FGC-practicing areas, where people would rather talk about women ‘*being members’* of Sande (i.e. a secret women-only society of ancestral origin which also operates in Guinea Conakry and Sierra Leone).

Whilst some midwives thought that it would be preferable to use the term *‘circumcision’* when working with Sande mothers in order to avoid *‘communication blocks’*, others thought that any comparison of FGC with male circumcision should be avoided. A few midwives explained how, according to the *‘traditional people’* (i.e. women members of the Sande society, and men members of Poro –its male equivalent), all women needed to have their clitoris cut because their clitoris could grow *‘as long as a penis’*. This was a traditional belief that one midwife defined as *‘a myth’*.

Other female genital practices that some Liberian women may engage in were described. Douching with locally-available substances to tight the vagina was mentioned. Half of the midwives reported having encountered some young women who, to provoke an abortion, had inserted intravaginally cassava, chalks and local herbs such as the locally-known as *‘rocket-propelled grenade’*. Three midwives reported that they had attended women who had died due to sepsis secondary to these intravaginal insertion for abortion practices.

### Highlighting the importance of becoming a Sande member

For the *‘traditional people’*, FGC was not only a procedure on the girls’ genitalia. All midwives confirmed that the FGC ceremony symbolized a girl’s entrance into Sande. Hence, according to most midwives, discussing FGC in public could be interpreted by the communities as questioning the legitimacy of Sande, the control that Sande exerts on the population, and the adequacy of the moral teachings that Sande endorses.

Sande membership was typically promoted by the elderly women, especially by the *‘zoes’* (i.e. the *‘zoe’* is the head of the Sande initiation camp; she is also known as *‘sowei’* in the literature [17]). The midwives explained how the *‘zoes’* used to organize and lead the Sande *‘bush’* (i.e. the initiation camp) every seven years approximately. The household males approved and provided resources to send their girls there. This was a tradition transmitted from generation to generation. Hence, most parents wanted their girls to join Sande in order to gain *‘respect’* from the community.

There was consensus that the main purpose of FGC was to remove the sexual desire of women. After the FGC ceremony, the girls entered a seclusion period where they would receive a series of *‘teachings’* aligned with the purpose of having initiated girls understand that women should inhibit their sex instincts. In this regard, some midwives explained how uninitiated women risked being perceived as *‘promiscuous’*, whilst, on the contrary, initiated women were trusted as *‘decent women’* by the community. Initiated women, trained by the *‘zoes’* to be better controlled by their parents and husbands, could *‘move freely’* around their communities. Additionally, as two midwives explained, due to the removal of sexual desire, FGC prevented women’s genital area from being *‘messy with fluids’* and feeling *‘itchy’*:

*They don’t have to be standing in front of people… because they feel that if the clitoris is there, like one told me: ‘it itches’. So, you don’t have to be among people and scratch it. So they [the women] feel fine without [the clitoris].*

All midwives agreed that Sande membership improved girls’ chances for marriage. A few recalled how, in the rural areas, during the celebrations that followed the end of the *‘bush’*, the men manifested interest in the initiated girls as future brides. The men appreciated that the importance of virginity and faithfulness had been emphasized by the *‘zoes’* during the *‘bush’*. In principle, as all girls had been *‘cut’* in the *‘bush’*, they were expected to be faithful because –as consequence of removal of sexual desire that was thought as main consequence of the excision of their clitoris– they would not need to seek for men other than their husbands to satisfy their own sexual drive.

Only one midwife described the *‘bush’* as a *‘rite of passage’* into womanhood. To all the other midwives, entering Sande –via the FGC ceremony– was not a prerequisite to becoming woman, but rather a prerequisite to gain the opportunity to being instructed to becoming *‘proper’*, *‘clean’*, *‘decent’* women that were to be perceived by the community as *‘fit to stay with a man’*. *‘Proper women’* were supposed to be *‘submissive’* women dedicated to serve their husbands, bear children for them, look after their husbands’ elders, and give them sex whenever they requested it. All midwives in this study manifested that the *‘zoes’s bush teachings’* were *‘good’*.

### Describing the FGC procedure

At the start of the Sande *‘bush’*, the girls were retained in a *‘special place’* that no midwife described in detail. When the day to perform FGC arrived, the girls were asked to sit in a bucket with water to *‘numb the area’.* The girls were then tied down, their hands held, and their legs separated. The *‘zoe’* excised the clitoris without any anesthesia. In the past, the *‘zoes’* used a *‘ritual knife’* prepared by a *‘country blacksmith’*. Nowadays, the *‘zoes’* may also use either an unsterilized razor blade or a surgical blade.

Pain, bleeding, and irreversible harm to adjacent tissues like the urethra were mentioned as the immediate harms of FGC. One midwife expressed that pain was so severe that it could lead to shock. Other midwife, a Sande member herself, stressed –drawing from her own personal experience– how profuse the bleeding could be:

*I can tell you. When I went there [to the ‘bush’, to be ‘cut’], I almost died. I bled almost to death*.

The midwives described how, to manage the bleeding, the *‘zoes’* applied local herbs. Should these local hemostatic remedies fail, the girls could die to hemorrhage. Some midwives admitted that, in the past, it was very unlikely that the *‘zoes’* could take the girl to a nearby clinic. A few argued that, even if the bleeding stopped thanks to these remedies, the unclean environment, the use of unsterilized instruments, and the substances used to clot could still lead to local infection in the genital area with potential to develop into sepsis.

The midwives described how, nowadays, the *‘zoes’* were using a single blade per girl. However, in the past, the same utensils were used to cut several girls. Some midwives believed that this was a possible cause of HIV and hepatitis transmission. No midwife recalled having ever dealt with any case of FGC-derived HIV and hepatitis acquisition in the clinics. Only one midwife mentioned that she witnessed a case of tetanus:

*I witnessed a tetanus case from that razor blade that they used. Because they used a blade that had been in the pack for so many months, and they feel it is a new blade, and they just went and used it.*

After the FGC ceremony, the girls had to stay secluded in the *‘bush’* to receive the *‘teachings’* from the *‘zoes’*. The seclusion period used to be as long as three years in the past. Nowadays, the seclusion period might have been reduced to 6 weeks. Allegedly, the *‘zoes’* obtained great *‘prestige’* out of their role as heads of the Sande initiation camps. In the past, they were compensated in kind with *‘lappas’* (i.e. local fabrics), rice, oil and beans. Some parents gave them money.

Nowadays, medical personnel may be invited to help prevent the immediate complications of FGC. One midwife admitted that she was invited to the *‘bush’* on various occasions. No midwife admitted having ever performed FGC themselves in the *‘bush’*. Only one midwife recalled how, in her clinic, she once *‘cut’* a pregnant woman who, right after delivery, requested her so. This midwife justified her act by stating that she used Lidocaine (i.e. an anesthetic drug) to *‘numb the place’*, which was a procedure that, according to her, the *‘zoes’* would not have done to that specific woman had she decided to go to the *‘bush’* instead.

### Unveiling the secret of FGC

The majority of midwives asserted that there was no benefit associated to FGC other than the *‘teachings’* that girls receive in the *‘bush’* after the *‘cutting’* ritual. In spite of no health benefit being associated to FGC, its rationale could not be publicly debated because everything related to the Sande society was deemed to be kept as a *‘secret’*. For Sande members, the *‘secret’* was enforced by an *‘oath’* sworn at the *‘bush’*. Fear to reprisals from the *‘zoes’*, who were described as *‘very fearful’* people, made Sande members afraid of conversing about FGC with non-members. One midwife proposed a visual metaphor: Sande members –same as the birds that never leave their flock– keep all their information, thoughts, needs and concerns within the Sande group:

*People would talk about it to members. There is secrecy around this thing. They say: ‘Birds of the same feathers fly together’. So, if people have to discuss something pertaining to [FGC], they won’t discuss it with just anybody.*

A consequence of the *‘secret’* was that the health implications of FGC were not discussed in midwifery school. Furthermore, in their workplaces, the *‘secret’* also affected midwife-patient relationships. All midwives explained that pregnant women would talk about their FGC state only to the midwives who were also Sande.

Besides respect for the *‘secret’*, both midwives and pregnant women could feel *‘shame’* to address FGC. Women themselves would *‘hide their initiation marks’* (e.g. razor-made cuts) in the consultation, and would sometimes blame previous *‘bad deliveries’* in order not to accept that their vulvar scars were the result of FGC. Acknowledgement of these feelings was described by most midwives as crucial for the establishment and maintenance of effective therapeutic relationships with pregnant women.

### Identifying obstetric consequences of FGC

All midwives described FGC as a harmful practice. However, there was no agreement on exactly which harms were directly associated to FGC. The only consensus reached was that many women experienced *‘tears’* from the (*FGC*) *‘scar’*, and *‘bleeding’* in delivery. The extent of the *‘tear’* and the *‘bleeding’* depended on the *‘size of the baby’s head’,* and on how the *‘scar’* impeded the vagina to *‘stretch’ during delivery*. According to the midwives’ estimates, the proportion of women suffering FGC-related complications in delivery could range from 20 to 60%, and such proportions would increase among adolescent mothers.

Due to secrecy, commonly, the midwives reported that they could only notice that the mothers had undergone FGC when performing vaginal examinations in antenatal care. Vaginal examinations were described as very useful to plan ahead the hygiene, asepsis, pain management, episiotomy and repair care needed to prevent harm during delivery. As an example, a few midwives explained how, in antenatal care, they could anticipate how to prevent the *‘tears’* by planning episiotomy.

Some midwives explained how, during delivery, the *‘tear’* could be impeded by *‘guiding the perineum’* or *‘holding together the* [FGC] *scar so it won’t split when the baby head passes’*, whilst the *‘bleeding’* from the *‘scar’* was usually stopped by exerting *‘pressure until clotting occurs’*. If unmanaged, a few midwives insisted that intra and postpartum hemorrhage could lead to death. However, no midwife reported any experience with a mother dying to FGC-attributable hemorrhage in labor.

*If you are dealing with somebody who is uncircumcised, you find that the person’s clitoris gives way to the fetus head. But a circumcised female has no flesh to give way. At the end of the day you will have huge laceration if you don’t do episiotomy! And it will give you plenty time [repairing] that particular woman. Because if you don’t repair [the tear], you will have that woman bleeding to death.*

Prolonged labor was identified by about half of the midwives as a direct consequence of FGC. Two midwives detailed how some women, due to the poor management of the *‘scar’* immediately after the *‘cutting’* ceremony*,* may present in antenatal care with the *‘whole vaginal orifice sealed up’*. These two midwives explain how women with such post-FGC vaginal orifice adhesions would be candidates for episiotomy or for delivery by caesarean, because they could suffer from obstructed labor, which could lead to laceration and bleeding.

Only a few midwives established that urinary tract infections could be observed as a result from the harm to the urethra during the FGC ceremony, and that fistula could result of FGC-induced obstructed labor. In fact, the majority of midwives disagreed that the risk of urinary tract infections, fistula, vulvar cysts and postnatal infections could be associated to FGC because, based on their experiences in the clinics, they argued that uncut women were also suffering from such complications.

The majority of midwives said that they would never talk to the mothers about the complications associated to FGC. To them, the mothers would disagree with their explanations for the following reasons: because mother could think as *‘offensive’* that the midwives dared to talk about a Sande ritual; because FGC was something that had happened to them *‘a long time ago’*; or because they might have already given birth to other children and might have not suffered any FGC-related harm in their previous deliveries.

### Identifying psychosocial consequences of FGC

The main purpose of FGC was to reduce sexual desire. Thus, many midwives claimed that they had encountered women who reported *‘lack of feelings for sex’*, and who complained of painful sex due to the vulvar *‘scar’* and to the FGC-related *‘tears’* suffered during their deliveries. Painful sex was described as especially worse for the first sexual intercourse for women of any age. Additionally, the midwives explained that –besides delivery– further laceration and bleeding could occur during vaginal intercourse, which would worsen sexual experiences for some women.

Some midwives expressed that they tried to explain to the mothers suffering from FGC-derived sexual impairment that sexual pleasure could be obtained from body areas other than the clitoris. Only one midwife, a Sande member herself, stated that she herself enjoyed sex because her husband did practice foreplay. To this midwife, FGC removed her desire for sex with men other than her husband, but it did not remove her capacity to obtain sexual pleasure. The other midwives did not mention anything about their own sexual lives.

Reportedly, the *‘traditional men’* (i.e. Poro members) were taught to expect that their wives would not feel sexual pleasure, would not demand sex from them, and would never deny them sex. Hence, a few midwives claimed that some men were *‘not sensitive’* to women’s sex problems. As one midwife explained, some men may *‘enjoy’* that the vagina of a *‘cut’* woman may be felt as *‘tigh*t*’* and *‘not messy with fluids’*. In addition, as another midwife explained, FGC was very *‘convenient’* to men in polygamous relationships. According to her narratives, if FGC were an effective procedure to remove women’s sexual desires, polygamous men would not need make much efforts to sexually satisfy all their wives:

*In the interior, you see one man having 3, 4, 5 wives. If [the clitoris] is not removed, the man won’t be able to satisfy all wives. [Each woman] would only go to the man three days [in a month]. After three days you [the man] are there [having sex with her every night], if you get [other] 10 women, then the 10 women would all [want to go and have sex after her] (Laughs)* (Excerpt adapted from colloquia).

Not all men were unaware of their partners’ problems. A few midwives explained how they had heard some Poro men complaining that their wives did not enjoy sex no matter how hard they tried to *‘stimulate’* them. One midwife said that some men may *‘jump out of the home’* to other *‘uncut’* women with whom, according to her, sex may be more satisfactory because such women would appreciate *‘the man’s efforts’*. Irrespective of the male partners’ attitudes towards women’s experiences with sex, psychosocial counseling in sex matters was described by all midwives as necessary for both male and female partners because *‘divorce’* and *‘problems in the home’* were reported as common consequences of FGC-attributable sexual impairment in women.

How refusal to enter Sande might cause social harm for women was also discussed during the interviews. Women who were not Sande and lived in FGC-practicing communities may not get married. Uninitiated women could receive names such as as *‘sinner’*, *‘offensive’*, or *‘dirt’*. The Sande members could ostracize non-members from routine social activities like conversing, praying and fetching wood. Many midwives agreed that suffering insults and isolation could make some adult uninitiated women take the decision to accept FGC as a prerequisite to enter Sande.

### Being trained on the health implications of FGC

Half of the midwives claimed that, when attending midwifery school, they acquired all necessary skills to manage FGC complications in delivery. Nevertheless, the majority said that they would appreciate further training on the potential risks associated to FGC; on why these risks were associated to FGC even if they could also be seen in *‘uncut’* women; on how to anticipate risks when performing visual examination of women’s genitalia in antenatal care; and on how to prevent harm from occurring in labor and delivery. Some noted that many registered midwives may be from non-Sande-operating counties and that, because they would not be *‘koronko’* (i.e. raised within Sande tradition), they might struggle when working in FGC-practicing communities. Additionally, as not all healthcare facilities had personnel skilled to attend women reporting psychosexual problems, the need to receive training to provide counselling was also remarked by most midwives:

*We just tell them that they have to cope with what they are going through right now because they cannot be repaired.*

Commonly, *‘traditional midwives’* (i.e. TBA) were perceived by the midwives as incapable to manage the FGC-related complications in delivery. Invariably, all midwives identified the TBA as *‘zoes’* or as Sande members. Training to the TBA to use antisepsis and hemostasis during the FGC ceremony was proposed. Nevertheless, the majority of midwives argued that training the TBA might be challenging. One midwife recalled having participated in a workshop on maternal health where the trainer *‘skipped’* the sessions on FGC to prevent the TBA leaving the room.

Some midwives, who had attended workshops on FGC expressed that the trainers made them feel *‘bad’* because they only emphasized the *‘negative side of it’* (i.e. the harmful health consequences) and did not talk about the *‘teachings’*. All midwives agreed that, for future trainings to effectively sensitize health care workers on the FGC-associated harms, these should incorporate content appreciating the Sande moral values and instructions given to the girls when in the *‘bush’.* A few also proposed that, if possible, FGC trainings should be facilitated by Sande health personnel.

In the past, the midwifery curricula did not include FGC due to the *secrecy* and because, as FGC was a *‘culture thought to be good’*, many health personnel failed to understand how its practice could cause ill health. The willingness to discuss FGC publicly may be changing. All midwives agreed that the midwifery curriculum should incorporate information on FGC.

### Engaging traditional representatives

Discussion of FGC at community-level, according to all midwives, must be planned very thoughtfully. Both female and male *‘zoes’*, the *‘elderly’* people, and *‘chiefs’* and *‘chairladies‘* would need to be involved as the traditional authorities who could facilitate community entry. A few midwives advised against *‘provoking’* the Sande-supported communities by openly advocating against FGC. There was consensus that, when reaching the traditional leaders with the aim to initiate community-level sensitization on the health implications of FGC, it would be convenient not to suggest them to discontinue the *‘bush’*. One midwife proposed the type of message that community mobilizers could convey to *‘convince’* the leaders to allow them reach the community members:

*“We respect the culture very much. We are part of it. But this part [FGC] causes death. We should have it optional. Let your children know that they will join the society. And let the health people be there to talk to them. If they want to, they will join. If they don’t want to, they should not be forced.”*

According to this and to other midwives, if *‘convinced’*, the leaders could grant permission to the mobilizers to talk about FGC to the community members; they could order the families to explain their girl children that they will have their genitalia excised in the *‘bush’* before joining Sande; they could ask the *‘zoes’* to either stop FGC or to substitute it by a *‘symbolic pricking’* of the clitoris; and they could also invite healthcare workers to the *‘cutting’* ceremonies to help prevent complications. There was consensus among the midwives that the perfect community mobilizer should be someone who knows the *‘zoes’*, with permission to talk to them on FGC and who is appreciative of the Sande *‘teachings’* and values. Preferably, a Sande member herself.

### Discussing politics

FGC was identified as a much contested issue in the political arena. As one midwife expressed, *‘that law must be passed’*. She was referring to the clause banning FGC in the domestic violence law that the *‘House of Representatives’* rejected in 2016 [30, 31]. All midwives discussed how the Members of the Liberian Parliament who rejected the clause might have been either Sande members themselves or non-members who were afraid of reprisal from the Sande (e.g. turning the electorate against them). The *‘zoes’* were defined as very influential on the community members’ electoral choices. Hence, according to all midwives, without the connivance of the *‘zoes’*, no law threatening the power of Sande could ever be approved at the Liberian Parliament.

Some midwives expressed concern that both the Ministry of Inner Affairs (LMIA) and the Ministry of Health and Social Welfare (MoHSW) were not *‘ready’* to support the abandonment of FGC. Reportedly, the LMIA was *‘still licensing zoes’* to organize the *‘bushes’*. Hence, when planning community mobilization, the LMIA needed to be involved as it had mandate to register the traditional authorities, and to regulate their activities.

Engagement of the MoHSW was also described as key to facilitate community mobilization. No midwife knew of any training, policy of campaign against FGC supported by the MoHSW. Most midwives thought that the MoHSW should support the conduct of FGC training facilitated by Sande members, at rural clinics-level. As one midwife put it, the MoHSW should ensure that *‘every midwife is educated on how to approach this particular* (Sande) *people’*.

### Disseminating and scaling-up FGC research

The midwives reflected on their participation as study subjects in this grounded theory research. The ones who were Sande explained that they had decided to talk about the society’s *‘secrets’* because they felt committed to the study aim. A non-member midwife expressed:

*I’m happy for this interview, because, as you interview others who have had some problems, it would show up. And then, based on those problems, we can now tender our curriculum, and training, and I hope that people will build communication skills that would help people discuss [FGC] in a way that is not offensive.*

The majority of midwives thought that dissemination of research findings in rural areas would be feasible if it were done in a *‘respectful manner’* and targeting the *‘proper’* people (i.e. *zoes*, leaders, the elderly). Dissemination in the healthcare sector was considered by the midwives as crucial to prevent maternal and children death because, as one midwife put it, many healthcare personnel may see as *‘normal’* the complications of FGC. Nevertheless, two midwifes thought that dissemination would not be feasible among the Sande and Poro healthcare workers.

Whilst the majority of midwives thought that dissemination would be feasible, only about half of them recommended scaling-up this research to include participants outside the healthcare sector. Only two midwives thought that it would be possible to talk to the women patients in the clinics about FGC. One midwife warned that extending this research at community-level could imply a high rate of refusals to participate. This midwife explained how some women would fear to be taken by force and be re-circumcised by the *‘zoes’* should these become aware of their participation as study subjects.

### Forecasting persistence of FGC

All midwives positioned themselves against FGC but in favor of the existence of Sande and, specially, in favor of the *‘teachings’* that the girls received in the Sande initiation camps. One midwife admitted that, though she would advocate for the abandonment of FGC, she actually thought that the practice was useful to discourage girls from engaging in early sex and having multiple partners:

*The bush school has a lot of good things. Even that genital mutilation is good in some way. Because, if your children are not running here and there, they will not be prostitutes […].*

Only one midwife, a non-member, said that she would *‘abolish’* Sande as well. The majority, though supportive of Sande, thought that FGC will *‘die of natural death’*. Some midwives mentioned that the rates of girls attending Sande already started to drop during the Liberian civil wars [32] and that the *‘bushes’* were not organized during the Ebola epidemic. In recent times, migration to urban areas contributed to women accessing formal education and the Internet and, hence, according to some midwives, many women could have learnt about other women’s health experiences with FGC and could have felt *‘empowered’* to decide not to have their daughters initiated into Sande. As one midwife put it, further investment in education was crucial to make people understand that *‘depriving a woman from her clitoris is a violation to her human rights’*.

Nevertheless, midwives members and non-members had a different perception of how the end to FGC could be achieved. Non-members emphasized that women who have suffered the consequences of FGC must have the responsibility *‘to stand up and say ‘no’ [to FGC]’* to their communities. Members tended to emphasize the role of leaders and *‘zoes’* to decide abandon its practice whilst continuing the *‘bush’*. The midwives members, who expressed that they had not subjected their own daughters to FGC, described themselves as educated professionals who had migrated to the urban areas and who were in a better socioeconomic position to make such decision. According to them, for women who remain in the rural areas to combat the social pressure to let their girl children be *‘cut’* in the Sande camps, a community mobilization strategy engaging traditional authorities since the outset was of utmost importance.

## Discussion

This study was a unique opportunity to frame how, in Liberia, improved access to information on FGM/C as a result of migration to urban areas and access to communication technologies; the societal control exerted by a secret female society; and the highly-valued significance of traditional gendered instructions to girls and women, interweave and determine the prospects for abandonment of FGM/C. This study shows how there are factors other than the FGM/C-associated health implications that shape prospects for its persistence or its abandonment in a country where any public discourse on FGM/C is discouraged either because of fear to reprisal from the Sande, or because people –including healthcare professionals– may consider FGM/C as an effective practice to keep women under control and compliant with socially-condoned gender norms.

Documenting that the FGM/C-attributable obstetric complications reported elsewhere [1, 5, 9, 33] are also encountered by midwives in Liberia calls for integration of FGM/C-tailored care into reproductive health services. Within the umbrella of possible maternal health strategies, FGM/C training to midwives deserves special consideration. Participants of this study have provided useful information on the cultural, political, and gendered nuances that determine how midwives look after their patients. Future FGM/C training could capitalize on this invaluable information.

The participants described how forced clitoridectomy, or FGM/C Type I, may lead to prolonged labor and to intrapartum vulvo-perineal laceration with subsequent severe bleeding. According to their narratives, many midwives –especially those born in non-FGM/C-practicing areas– lack skills to properly perform vaginal examinations to Sande women during antenatal care; to anticipate possible FGM/C-related harms in delivery; and to plan ahead prevention of complications during delivery. University-based and continuous professional education on the identification and management of FGM/C-related complications should be promoted. Since many midwives work in relative isolation and lack immediate access to health training resources, mobile health applications such as ‘Let’s talk FGM’® could be adapted for use by Liberian midwives in remote rural areas [34]. As demonstrated in a recent study in United Kingdom [8], tailored FGM/C training for midwives could contribute to improve maternal health. In that study, FGM/C was surprisingly not associated with increased incidence of adverse obstetric complications. The authors concluded that this finding related to the investment made in training to the clinical staff.

In agreement with narratives of women participants in other FGM/C studies in West Africa [35, 36], FGM/C is done to eliminate women’s sexual desire and prevent pre- or extramarital sex. In Liberia, traditionally, the community expects women to be sexually *‘submissive’* to their partners. The midwives participants recognized that Liberian women’s problems with sex or intimacy are being left unaddressed due to the secrecy that surrounds FGM/C, and also due to lack of midwives’ counselling capacities. Interestingly, in spite of common concern on women’s sexual wellbeing, all midwives claimed that they appreciated the *‘bush teachings’* that reinforce gender roles placing women as subjected to men’s sex needs. No midwife expressed that these gendered norms should be reframed or contested. Indeed, as suggested by the participants, sexual counselling provision must be a component of FGM/C training to midwives. However, for Sande women to achieve sexual health, further debate and promotion are needed –at societal-level– on women’s sexual rights to have respect for bodily integrity; choose their partners; decide to be sexually active or not; and pursue a pleasurable sexual life.

During the informed consent process, all midwives participants were assured that no question on Sande was to be posed. Nevertheless, all voluntarily decided to talk about Sande during the interviews. This attitude was especially determined by their perception of anti-FGM/C interventions as not being appreciative of Sande. All but one claimed that FGM/C needs be stopped, but that young girls need to continue receiving the *‘bush teachings’*. As a result of the midwives’ willingness to share their knowledge beyond their experiences in their consultations, this study contributes with information on the Sande society that may be extremely useful to inform culturally-sensitive advocacy for abandonment –or replacement by another symbolic non-harmful procedure– of FGM/C as a prerequisite to Sande membership.

Nevertheless, careful consideration needs to be given when designing advocacy for abandonment of FGM/C as this practice in Liberia is a sensitive political issue. To our knowledge, this situation is unique in sub-Saharan Africa, save for neighboring Sierra Leone and Guinea Conakry [16, 17, 19, 21]. In this region, FGM/C symbolizes women’s ascription to an institution with ancient historical roots that holds significant economic, social and legislative power [16, 17, 21, 37]. As explained by the study participants, there might be some Members of the Parliament that would not risk lose the trust of the most *‘traditional’* sector of their electorate (i.e. Sande and Poro members) by voicing their personal standpoints against FGM/C. The result is that, in Liberia, there is not much room for public discussion on FGM/C. This situation in similar to Sierra Leone, where, according to Mgbako et al., people may *‘view attacks on FGM as attacks on the Bondo (i.e. Sande)’* [21].

In the Liberian media, there are reports of threats of physical harm as punishment for breaking the *‘oath’* of silence on FGM/C that all girls must make during the Sande initiation rituals [38, 39]. FGM/C is a prized symbol of belonging to Sande. But it is also a feared symbol of punishment (i.e. circumcised by force [38]). Fear to being harmed prevents Sande members from abandoning the society, and from sharing any information on its rituals with non-members. Today, however, whilst all participants were not optimistic about the possibility of engaging the traditional communities in FGM/C awareness, they explained how some Liberians are changing their attitudes towards FGM/C. They described a societal change that was not promoted by any anti-FGM/C intervention they held any knowledge of. The change was dramatically initiated by the civil wars and by the Ebola outbreak, events that displaced people from the rural to urban areas and that made difficult the celebration of the Sande initiation camps. Today, Liberians are easily accessing education, mass media and the Internet. Allegedly, many women are self-learning, without the governments’ aid, on the FGM/C-attributable harms, and are taking an informed decision to abandon it. Exposure to the global world threatens the power of Sande to control the sharing of information on their initiation rituals. In this scenario, health authorities must map the structural and contextual factors that may promote the abandonment of FGM/C. These factors can be used to plan FGM/C integrated maternal health interventions that would challenge harmful gender norms and that, at the same time, would be sensitive enough to avoid offending Sande members. As emphasized in this study, there are still many Liberian women in the rural areas with limited access to education opportunities and to information technologies. These women need be empowered to be able to debate in their communities about the persistence of FGM/C, and to negotiate its absolute abandonment or its transformation in non-harmful symbolic forms (i.e. *‘pricking’*).

### Strengths and limitations

A strength of this study was the application of a feminist interpretation of constructivist grounded theory. The participants were addressed as co-interpreters of the findings. They were motivated to identify issues of oppression for women and midwives (e.g. forced silence on FGM/C and Sande, gender norms harmful to girls and women), and to propose measures to address them.

Nevertheless, several limitations impeded the smooth conduct of this study. Firstly, three approached midwives, all Sande members, refused to enrol. Hence, we might have missed viewpoints from midwives who favored FGM/C. The sampling method that we used might have also made us miss opportunities to encounter other pro-FGM/C midwives. Secondly, though we aimed to interview TBA, we finally desisted because the participants discouraged us. As per their narratives, all TBA are *‘zoes’*. It needs to be noted that this could not be corroborated by the research team. In the same line, though focus group discussions should have been organized to share our interpretations from the individual interviews’ data, neither the participants nor the research team felt that it was safe to discuss FGM/C in a group. Additionally, peer-checks with healthcare workers other than the participants were not advised. All these limitations made triangulation difficult.

Early discontinuance of recruitment did not impede us to generate sufficient valid data to meet the study objectives. Unfortunately, early discontinuance did prevent us from reaching theoretical saturation in some crucial aspects (e.g. possible medicalization of FGM/C in Liberia), and from exploring in depth some emergent topics, such as what role the community of Liberians living in United States may be playing in the persistence of Sande.

## Conclusion

FGM/C symbolizing a woman’s entrance to a secret female society with widely perceived social benefits, deters politicians, health authorities and the community at large to promote and participate in public interventions aiming at its eradication. However, migration to urban areas and improved access to information and communication technologies might be fuelling abandonment of this harmful practice. Irrespective of FGM/C seemingly being in the decline, surveillance is warranted at clinic-level to prevent medicalization. Liberian midwives need training to provide sexual counselling and to attend the obstetric needs of pregnant women who underwent FGM/C. Measures to prevent FGM/C-related complications during delivery need be standardized and implemented in all healthcare facilities. Any training and community engagement targeting FGM/C-practicing populations need be designed in collaboration with Sande members; thus taking into utmost consideration that, although many may disapprove of FGM/C, the majority of the population may appreciate the traditional values and norms that have been transmitted to Liberian girls from generation to generation. In this scenario, the possibility of replacing FGM/C by non-harmful symbolic procedures as ritual prerequisite for Sande membership could be explored with FGM/C-affected communities.

## Abbreviations

FGC: Female Genital Cutting
FGM/C: Female Genital Mutilation/Cutting
IDI: In-depth Interviews
LISGIS: Liberia Institute of Statistics and Geo-Information Services
LMIA: Liberia Ministry of Inner Affairs
MoHSW: Ministry of Health and Social Welfare
OHCHR: Office of the United Nations High Commissioner for Human Rights
TBA: Traditional Birth Attendants
WHO: World Health Organization

## References

[1] World Health Organization (WHO). Care of girls & women living with female genital mutilation. A clinical handbook. Geneva: World Health Organization; 2018.

[2] Abdulcadir J, Botsikas D, Bolmont M, Bilancioni A, Djema DA, Bianchi Demicheli F (2016). Sexual anatomy and function in women with and without genital mutilation: a cross-sectional study. J Sex Med.

[3] Smith H, Stein K (2017). Psychological and counseling interventions for female genital mutilation. Int J Gynaecol Obstet.

[4] Okomo U, Ogugbue M, Inyang E, Meremikwu MM (2017). Sexual counselling for treating or preventing sexual dysfunction in women living with female genital mutilation: a systematic review. Int J Gynaecol Obstet.

[5] World Health Organization (WHO) (2016). WHO guidelines on the management of health complications from female genital mutilation.

[6] Gayle C, Rymer J (2016). Female genital mutilation and pregnancy: associated risks. Br J Nurs.

[7] Valor N, Dawson A, Turkmani S, Hall JJ, Nanayakkara S, Jenkins G (2016). Obstetric outcomes for women with female genital mutilation at an Australian hospital, 2006-2012: a descriptive study. BMC Pregnancy Childbirth.

[8] Balachandran AA, Duvalla S, Sultan AH, Thakar R (2018). Are obstetric outcomes affected by female genital mutilation?. Int Urogynecol J.

[9] World Health Organization (WHO). Female genital mutilation and obstetric outcome (2016). WHO collaborative prospective study in six African countries. Lancet.

[10] Johansen REB, Diop NJ, Laverack G, Leye E (2013). What works and what does not: a discussion of popular approaches for the abandonment of female genital mutilation. Obstet Gynecol Int.

[11] Shell-Duncan B (2001). The medicalization of female “circumcision”: harm reduction or promotion of a dangerous practice?. Soc Sci Med.

[12] Christoffersen-Deb A (2005). “Taming tradition”: medicalized female genital practices in Western Kenya. Med Anthropol Q.

[13] Umar AS, Oche MO. Medicalization of female genital mutilation among professional health care workers in a referral hospital, North-Western Nigeria. J Reprod Biol Health 2014;2(2):1–7.

[14] Muthumbi J, Svanemyr J, Scolare E, Temmerman M, Say L (2015). Female genital mutilation: a literature review of the current status of legislation and policies in 27 African countries and Yemen. Afr J Reprod Health.

[15] Liberia Institute of Statistics and Geo-Information Services (LISGIS), Ministry of Health and Social Welfare, National AIDS Control Program, ICG International.. Liberia. Demographic and Health Survey. 2013. Monrovia: LISGIS; 2014.

[16] Dapper O (1964). Umbständliche und Eigentliche Beschreibung von Afrika anno 1668.

[17] Richards JVO (1975). Some aspects of the multivariant socio-cultural roles of the Sande of the Mende. Can J Afr Stud.

[18] Ahmadu F, Shell-Duncan B, Hernlund Y (2000). Rites and wrongs: an insider/outsider reflects on power and excision. Female “circumcision” in Africa: culture, controversy and change.

[19] Berliner D, Argenti N, Schramm K (2012). Memories of initiation violence: remembered pain and religious transmission among the Bulongic (Guinea, Conakry). Remembering violence. Anthropological perspectives on intergenerational transmission.

[20] Bjälkander O, Grant DS, Berggren V, Bathija H, Almroth L (2013). Female genital mutilation in Sierra Leone: forms, reliability of reported status, and accuracy of related demographics and health survey questions. Obstet Gynecol Int.

[21] Mgbako C, Saxena M, Cave A, Farjad N, Shin H (2010). Penetrating the silence in Sierra Leone: a blueprint for the eradication of female genital mutilation. Harvard Human Rights J.

[22] Liberia Institute of Statistics and Geo-Information Services (LISGIS), Ministry of Health and Social Welfare, National AIDS Control Program, Macro International Inc. Liberia. Demographic and Health Survey. 2007. Monrovia: LISGIS and Macro International Inc.; 2008.

[23] Kaplan A, Hechavarría S, Bernal M, Bonhoure I (2013). Knowledge, attitudes and practices of female genital mutilation/cutting among health care professionals in the Gambia: a multiethnic study. BMC Public Health.

[24] Dolo O, Clack A, Gibson H, Lewis N, Douthall DP (2016). Training of midwives in advanced obstetrics in Liberia. Bull World Health Organ.

[25] Luginaah IN, Kangmennaang J, Fallah M, Dahn B, Kateh F, Nyenswah T (2016). Timing and utilization of antenatal care services in Liberia: understanding the pre-Ebola epidemic context. Soc Sci Med.

[26] Lori JR, Danielson Rominski S, Perosky JE, Munro ML, Williams G, Anne Bell S (2015). A case series study on the effect of Ebola on facility-based deliveries in rural Liberia. BMC Pregnancy and Childbirth.

[27] Plummer M, Young LE (2010). Grounded theory and feminist inquiry: revitalizing links to the past. West J Nurs Res.

[28] Martínez Pérez G, Mubanga M, Tomás Aznar C, Bagnol B (2015). Grounded theory: a methodology choice to investigating labia minora elongation among Zambians in South Africa. Int J Qual Methods.

[29] Wuest J (1995). Feminist grounded theory: an exploration of the congruency and tensions between two traditions in knowledge discovery source. Qual Health Res.

[30] Johnson-Mbayo B. Liberian women protest for passage on domestic violence bill. In: Frontpage Africa online. 2017. https://allafrica.com/stories/201705170862.html Accessed 10 Feb 2019.

[31] Wandia M. Liberia needs to muster the courage to ban FGM. In: The Guardian. 2016. https://www.theguardian.com/global-development/2016/apr/27/liberia-courage-to-ban-fgm-ellen-johnson-sirleaf. Accessed 10 Feb 2019.

[32] Lidow NH (2016). Violent order: understanding rebel governance through Liberia’s civil war.

[33] Adam T, Bathija H, Bishai D, Bonnefant Y, Darwish M, Huntington D (2010). Estimating the obstetric costs of female genital mutilation in six African countries. Bull World Health Organ.

[34] McEwan J (2016). Let’s talk FGM’: An app to support professionals and clients discuss a difficult subject.

[35] Iliyasu Z, Abubacar IS, Galadanci HS, Haruna F, Aliyu MH (2012). Predictors of female genital cutting among university students in northern Nigeria. J Obstet Gynaecol.

[36] Smith C (2008). Creating spaces: challenging conventional discursive norms surrounding the marking of women’s bodies. Finnish Journal of Ethnicity and Migration.

[37] Mendelson EM, MacKenzie NI (1968). Primitive secret societies. Secret societies.

[38] Batha E. Kidnappers jailed for forcing Liberian woman to undergo FGM. In: Trust Thomson Reuters Foundation; 2013. http://news.trust.org/item/?map=kidnappers-jailed-for-forcing-liberian-woman-to-undergo-fgm. Accessed 10 Feb 2019.

[39] Batha E. Secret societies make Liberia one of the hardest places to end FGM. In: Trust Thomson Reuters Foundation; 2014. http://news.trust.org//item/20140205144950-niqxw/. Accessed 10 Feb 2019.

